# Seasonal Fluctuations and Vertical Heterogeneity of Biochemical–Structural Parameters in Wetland Emergent Aquatic Vegetation

**DOI:** 10.34133/plantphenomics.0275

**Published:** 2024-11-28

**Authors:** Huaijing Wang, Yunmei Li, Jianguang Wen, Gaolun Wang, Huaiqing Liu, Heng Lyu

**Affiliations:** ^1^ State Key Laboratory of Remote Sensing Science, Aerospace Information Research Institute, Chinese Academic of Sciences, Beijing 100101, China.; ^2^ Key Laboratory of Virtual Geographic Environment of Education Ministry, Nanjing Normal University, Nanjing 210023, China.; ^3^ Jiangsu Center for Collaboration Invocation in Geographical Information Resource Development and Application, Nanjing 210023, China.; ^4^ University of Chinese Academic of Sciences, Beijing 100049, China.

## Abstract

Accurate understanding of vertical patterns of canopy structure characteristics and solar radiation distribution patterns of aquatic vegetation is pivotal in formulating a bidirectional reflection model and comprehending the ecological dynamics of wetlands. Further, physiological and biochemical stratified structural properties of aquatic vegetation in wetlands remain unexplored due to more inherent investigation challenges than terrestrial vegetation. This study evaluated the structural characteristics of vegetation communities and the regulation of direct solar radiation variations within the canopy across seasons of *Phragmites australis (P. australis)* and *Typha orientalis (T. orientalis)*, 2 typical emergent aquatic vegetations (EAVs), based on radiative transfer theory. Observations revealed that physiological and biochemical metrics varied at different growth stages with canopy height, the stratified leaf area index in the middle being higher than at the top and bottom of the *P. australis* cluster. Moreover, the vertical profiles of direct solar radiation decrease with depth, showing a bowl-shaped and V-shaped curve in the *P. australis* and *T. orientalis* clusters, respectively. Interestingly, the sensitivity of layered solar direct radiation transmittance to canopy structural parameters is obviously higher than that of canopy pigments, suggesting considerable potential for estimating layered structural parameters. The transmittance of direct solar radiation decreases with increasing leaf area index at different heights, and stratified transmittance in the cluster can be accurately described by a negative binomial function with a deviation of less than 2%.

## Introduction

Wetlands are globally distributed, variable ecosystems offering numerous ecological, economic, and societal advantages. Wetlands store approximately 30% of global soil carbon but cover only 3 to 5% of the planet [1–4]. Emergent aquatic vegetation (EAV), or macrophytes, is a typical aquatic vegetation in wetlands and a key primary producer in shallow waters, and is an essential component of transitional environments between inland freshwater and coastal ecosystems [5–8], due to its role in slowing water flow, attenuating wave energy, capturing particulate matter [9], and promoting sediment oxygenation [10–13]. Therefore, understanding the ecological processes of aquatic vegetation in wetlands is of paramount importance. This requires understanding the physiology, biochemistry, and dynamic alterations of aquatic plant communities, especially their vertical differentiation patterns of pigment systems and community structures. Recently, scholars have studied the impact of vertical heterogeneity distribution patterns of leaf inclination angle [14], leaf shape, clumping index [14], and equivalent water thickness [15–17] of terrestrial vegetation on microclimate and climate, especially under global change background. Scholars generally focus on exploring how these vertical variations affect canopy light [18–20], water, nutrient absorption, and photosynthetic rate. Recent research has found that these structural factors influence plant growth and phenology by adjusting canopy radiation and temperature [21]. The seasonal variation trend of biochemical and structural parameters across the vertical direction of wetland aquatic vegetation reveals ecological processes during the growth of aquatic vegetation. Compared to terrestrial ecosystems, the understanding of biochemical changes in wetland ecosystem, especially vertical profiles of aquatic vegetation, is very limited [22].

Aquatic vegetation intercepts radiation from the sun for photosynthesis and organic matter accumulation, which is governed by the canopy structure and pattern of radiation distribution within the canopy [23,24]. Specifically, leaf shape, leaf normal inclination angle distribution (LAD) [25], leaf area index (LAI), and especially vertical distribution patterns influence canopy radiation interception. Also, canopy extinction coefficient, leaf projection function, and solar incident angle affect this process [26]. Furthermore, special underlying surfaces of aquatic vegetation, such as water, sediment, and algal [27], result in variations in solar radiation distribution patterns in aquatic vegetation clusters [23], significantly impacting canopy reflectance and solar radiation fields within the canopy. This requires plant reconfiguration in biomass allocation strategies and leaf morphologies to meet light energy requirements.

The physical remote sensing model-based method is expected to quantify stratified physiological, biochemical, and structural properties of wetland aquatic vegetation. Studying solar radiation transmittance in plant communities provides crucial insights into the bidirectional reflectance model of aquatic vegetation communities. Is there a potential relationship between these stratified biochemical–structural and light radiation parameters to retrieve vertical profiles of biochemical structural parameters? Two typical EAV *Phragmites australis* (*P. australis)* and *Typha orientalis (*T. orientalis*)* in wetlands were selected as case studies, based on ground observation experiments over their entire growth cycle to simulate and reveal (a) biochemical–structural vertical characteristics within the canopy (e.g., the leaf normal inclination angle, leaf shape, and LAI), (b) layered canopy light interception characteristics (leaf projection function and canopy extinction coefficient), and (c) distribution and simulation of direct solar radiation transmittance within the canopy.

## Materials and Methods

### Study site and field observation

#### Study site

**Fig. 1.**
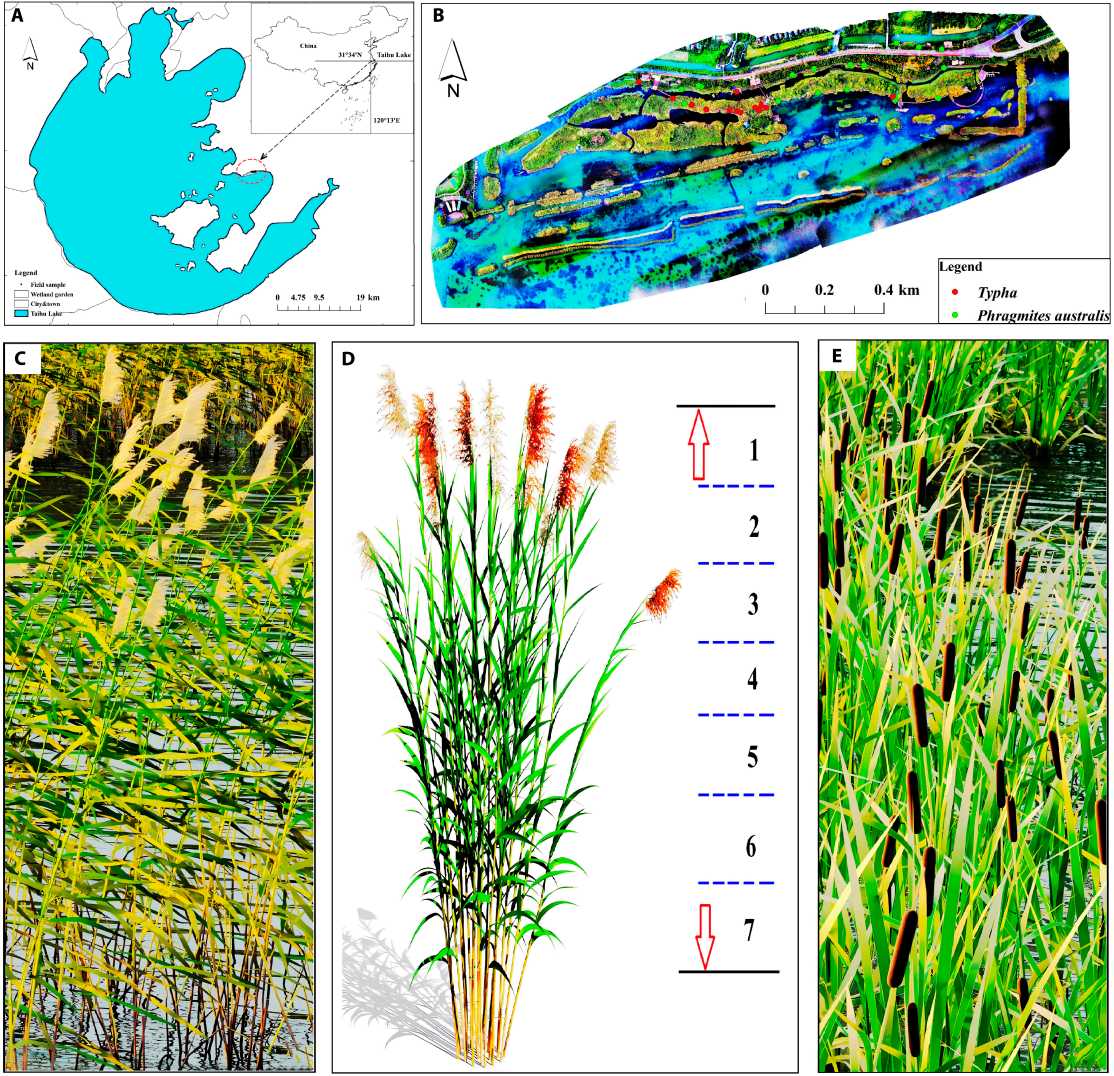
Study area and distribution of in situ measurements. (A) Map depiction of Taihu Lake’s geographic scope in China. (B) Localization map for Taihu National Wetland Park, exhibiting sampling sites for aquatic vegetation within the wetlands, overlaid with an RGB image from DJ Phantom 3 UAV, May 2021. (C) Canopy photo of *P. australis*. (D) Layered scheme for measuring biochemical parameters of canopy leaves. (E) Canopy photo of *T. orientalis*.

The Taihu National Wetland Park is located on the eastern shore of Xukou Bay (longitude: 120.41°E to 120.44°E; latitude: 31.21°N to 31.2°N), bordering the northern boundary of the subtropics. It has distinct seasons, abundant precipitation and sunlight, and a pronounced monsoon climate pattern. The Wetland Park was designed and completed in 2005. To showcase the park, an artificial island was constructed through sediment excavation and river channel dredging for ecological conservation purposes. *P. australis* (Fig. 1D) and *T. orientalis* (Fig. 1E) were planted in blocks to enrich the aquatic wetland environment throughout the island.

*P. australis* and *T. orientalis* are ubiquitous perennial aquatic or marshy herbs showing robust environmental adaptation, with their vigorous growth phase from April to June and maximum biomass from July to October [28,29]. Under the regulation of water levels and buffering of lakeshores, aquatic vegetation within Wetland Park is almost unaffected by external disturbances under horticultural management schemes. A study was conducted in the park from 2020 to 2022 to monitor the vertical structure of the canopy and its variations across seasons (spring: March to May; summer: June to August; autumn: September to November; winter: December to February of the following year), as shown in Fig. 1.

#### Field observation

In situ investigation of structural parameters of the EAV canopy was facilitated by 31 permanent sample plots, 18 for *P. australis* and 13 for *T. orientalis*. To ensure consistent growth observation and uniform sampling, each sample was positioned within a 2 × 2 m^2^ experimental plot, divided into 4 equal square areas of 1.0 m^2^ by using colored nylon tape. Fixed sample plots were marked for destructive sampling over 4 seasons to ensure accurate measurement results in aquatic environments.

The LAD of aquatic vegetation was considered a species-specific trait [30–33]. On 2020 September 12 [day of the year (DOY) = 256], 2020 December 21 (DOY = 355), 2021 May 9 (DOY = 129), 2021 October 28 (DOY = 301), and 2022 July 12 (DOY = 193), the photographic technique was employed to quantify a substantial number of leaf normal inclination angles for a single plot of each species. Throughout the observation period, the mean canopy height of *P. australis* was 2.45, 3.61, 4.64, and 4.82 m, respectively. Each quadrat was evenly divided into 7 sublayers from top to bottom of the canopy. Canopy is just evenly divided into 3 layers to ensure statistical significance and balance workload when measuring and analyzing the vertical distribution pattern of the leaf normal inclination angle [34].

Upon measuring leaf spectral properties, several leaf characteristics were quantified in the scientific laboratory: leaf chlorophyll concentration $\left(C_{\mathrm{ab}},g/{\mathrm{cm}}^{2}\right)$, leaf xanthophyll concentration ($C_{\text{brown}},g/{\mathrm{cm}}^{2}$), fresh weight (FW,g), and dry weight (DW,g). Leaf surface area ($\mathrm{LA},{\mathrm{cm}}^{2}$) was scanned by using an AMH 350 area meter. Subsequently, leaf water content (LWC,g/g), equivalent water thickness (EWT,cm), leaf mass area ($\mathrm{LMA},g/{\mathrm{cm}}^{2}$), and specific leaf area ($\mathrm{SLA},{\mathrm{cm}}^{2}/g$) were calculated from fresh weight and leaf area, as described by Gara et al. [35].

After measuring the leaf inclination angle, the maximum width and length of all the leaves were quantified with a millimeter ruler for each square site. The area of individual leaves was predicted by an empirical regression model that was confirmed to be reliable in our previous papers [34]. Ultimately, the leaf area for each layer and the LAI of the plot were calculated through statistical analysis and unit conversion.

### Method

The biochemical–structural parameters and solar radiation were analyzed to reveal the vertical distribution pattern within the canopy, such as pigment, LAD, LAI, canopy projection function, and extinction coefficient. Seasonal dynamics of these parameters provide insights into growth and ecological processes.

#### Canopy structure and light radiation

The leaf normal inclination angle denotes the angle between the leaf surface and the normal direction of leaf surface. The trigonometric algorithm was confirmed to be the best fit for leaf angle distribution based on our previous study [34,36]. The leaf projection function $G\left(\theta \right)$ depicted the absorption, reflection, or transmission of solar radiation by individual leaves within a canopy. Given an azimuthally symmetric canopy, the leaf projection function $G\left(\theta \right)$ quantifies the unit leaf area projection coefficient on a plane perpendicular to the viewing direction [30,36,37]. The canopy extinction coefficient (K) explains how much light is attenuated by the leaf and other plant organs. Further details are provided in [38,39] and Appendix A.

The direct solar radiation transmittance of the canopy assesses the percentage of incident sunlight that penetrates the canopy and reaches the ground, and the transmittance at a specific depth indicating the remaining proportion of direct light at that depth. The direct solar radiation transmittance of canopy is quantified by a given LAI based on Beer–Lambert law [40–42], which is expressed as:

$$T_{s}=e^{\left(-L*\frac{G\left(\theta ,\theta_{L}\right)}{\text{cos}\left(\theta \right)}\right)}=e^{\left(-L*K\right)}$$ (1)

$T_{s}$ denotes the direct solar radiation transmittance, *L* signifies the LAI, $\theta_{L}$ represents the leaf inclination angle, and θ is the view zenith angle of the sun.

Furthermore, evenly dividing the canopy into *N* horizontal layers allows us to quantify that direct solar radiation is intercepted by each sublayer. Canopy clusters are equally divided into *N* layers (Eq. 2) and statistically independent in this study. To depict the vertical distribution of direct solar light within each layer with LAI, we employ negative binomial functions (Eq. 3) to simulate the layered direct solar radiation transmittance and the layered direct solar radiation transmittance (Eq. 4), described as follows:

N=L/∆L (2)

$$T={\left(1+K*∆L\right)}^{-\frac{L}{∆L}}$$ (3)

$$T_{i}={\left(1+\frac{K}{N}\right)}^{-i},\text{where}\;i=1,2,\cdots \cdots N$$ (4)

where *K* is the canopy extinction coefficient and T and $T_{i}$ represent direct solar transmittance and layered direct solar transmittance, respectively.

#### Performance budget

Referring to Weiss et al.’s [33,43] approach, ε and δ, 2 critical metrics were applied to evaluate the accuracy and bias of the simulation model. Calculation of these indicators is outlined below:

$$\epsilon =100*\left(10^{Y}-1\right)\left[\%\right],\text{where}\;Y=\text{Median}\left|{\text{log}}_{10}\left({\tilde{y}}_{i}/y_{i}\right)\right|$$ (5)

$$\begin{array}{c} \delta =100*\mathrm{sign}\left(Z\right)\left(10^{\left|Z\right|}-1\right)\left[\%\right],\text{where}\;Z\\ \;=\text{Median}\left({\text{log}}_{10}\left(\frac{{\tilde{y}}_{i}}{y_{i}}\right)\right) \end{array}$$ (6)

Here, $y_{i}$ and ${\tilde{y}}_{i}$ denote the simulated value and the true value, respectively. The Median operator was applied to convey positive or negative deviation when calculating ϵ (median symmetric accuracy) and δ (signed percentage bias). Smaller ϵ and δ mean higher accuracy and smaller deviation, which means better consistency between measurement and simulation.

Furthermore, the *R*^2^ metric validates the reliability of models.

$$R^{2}=1-\frac{\sum_{i}{\left({\tilde{y}}_{i}-y_{i}\right)}^{2}}{\sum_{i}{\left({\bar{y}}_{i}-y_{i}\right)}^{2}}$$ (7)

where ${\tilde{y}}_{i}$ denotes the estimated value by the model and ${\bar{y}}_{i}$ and $y_{i}$ represent the mean and measured values of the target variable, respectively.

## Results

Based on data collected from 2020 to 2022 in Taihu Lake National Wetland Park, this study conducted a detailed analysis of mean projection and extinction coefficient in relation to changes in direct solar radiation transmittance within the EAV canopy cluster. These results provide valuable insights for constructing bidirectional reflectance models for aquatic vegetation.

### Architecture characteristics of canopy

**Fig. 2.**
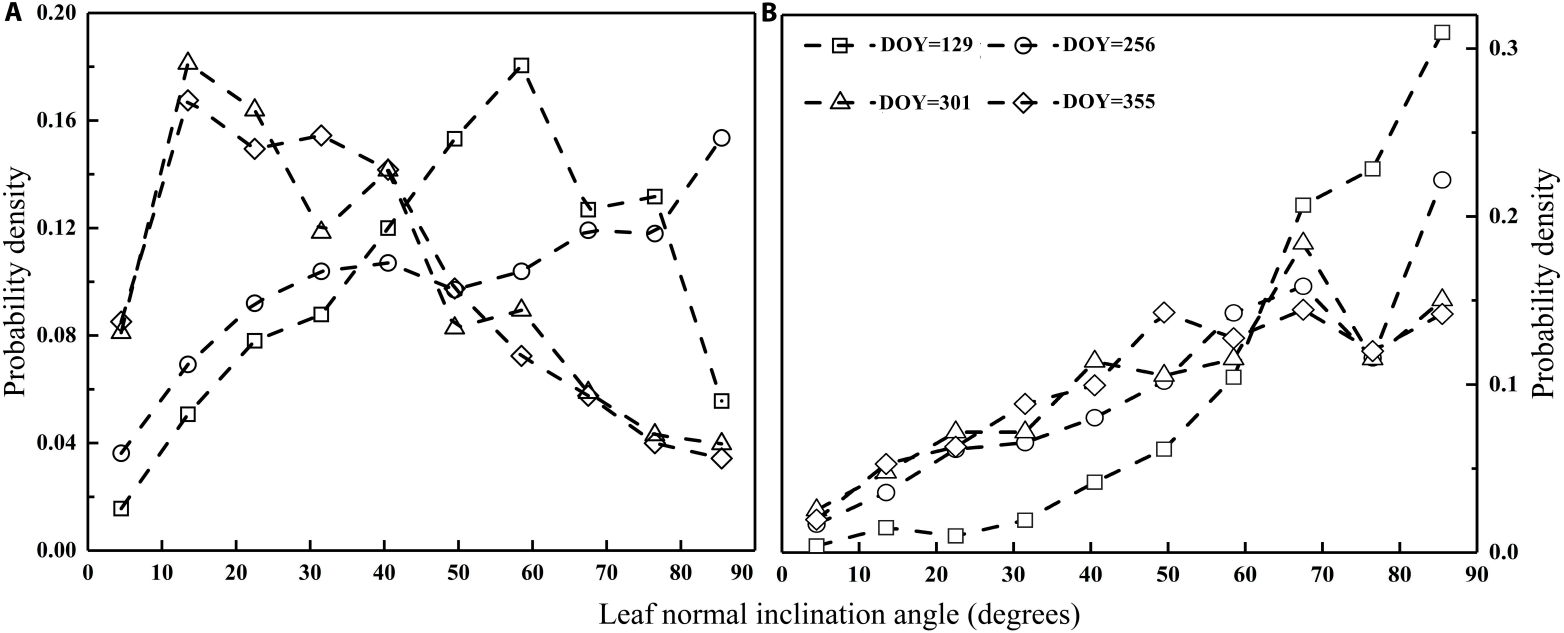
Distributional probability densities of leaf inclinations, depicted in (A) *P. australis* and (B) *T. orientalis*. Leaf normal inclination angle equal to 90° denotes a vertical inclination.

**Fig. 3.**
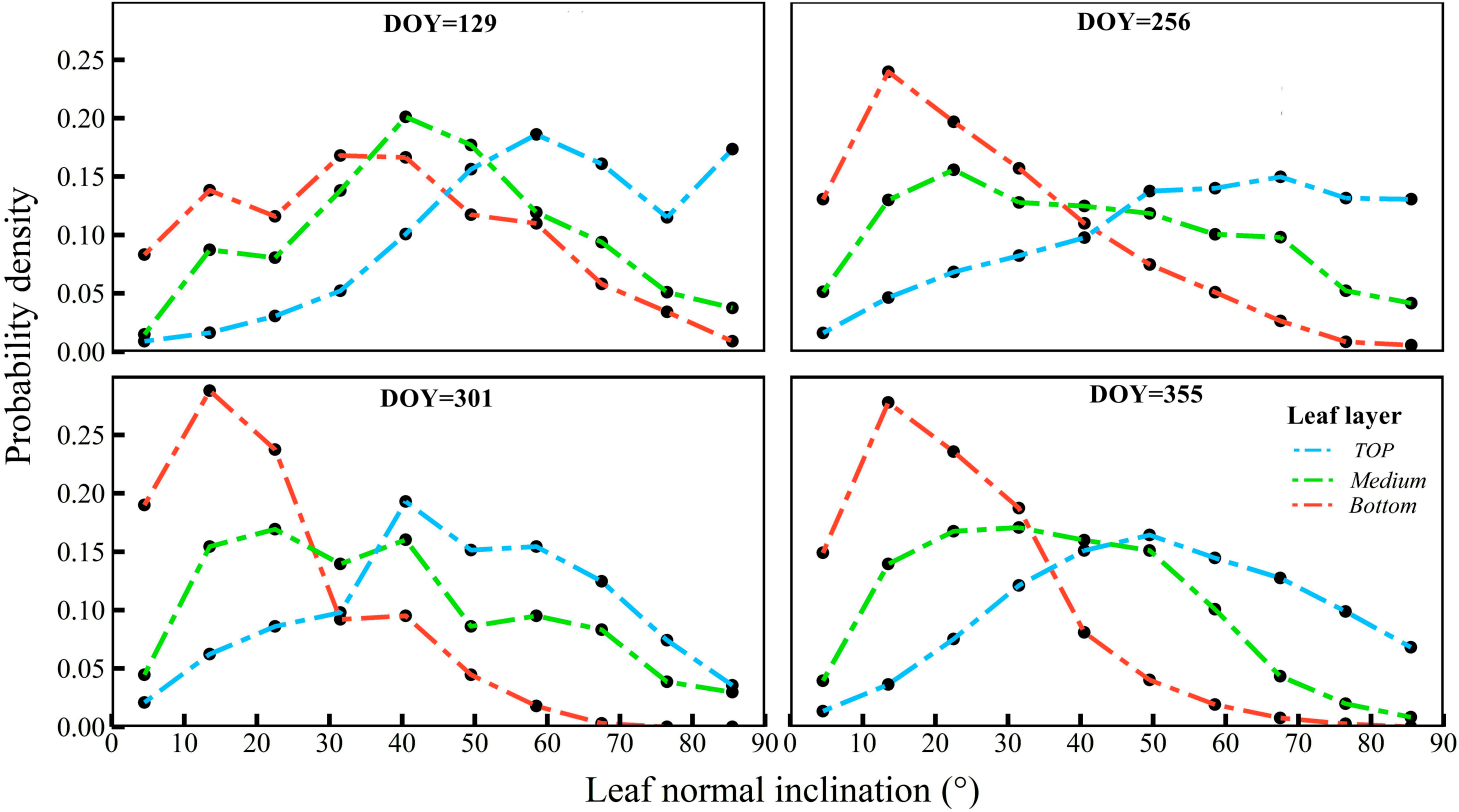
Probability density of *P. australis* leaf distribution along canopy height. A leaf normal inclination angle of 90° denotes a vertical leaf.

According to the photographic method, the leaf normal inclination angle of typical aquatic vegetation species over the 4 seasons (Fig. 2) and along the canopy height (Fig. 3), *P. australis* and *T. orientalis*, was calculated by trigonometry algorithm described in Appendix A.

#### Seasonal variation of leaf normal inclination angle

Continuous field observations revealed that the LAD of *P. australis* significantly fluctuates with the seasons, resembling erectophile, erectophile, planophile, and planophile from spring to winter, respectively, as described by Goel [44] (Fig. 2). The LAD of *P. australis* in spring and summer parallels an erectophile pattern, indicating a higher proportion of upright leaves during this period. However, the pattern shifts dramatically to the planophile pattern in autumn and winter, suggesting an increase in the proportion of horizontal leaves, potentially enhancing the light energy captured by the canopy (Fig. 2).

However, the leaf normal inclination of *T. orientalis* exhibits a unique feature, with the LAD always erectophile pattern throughout its growth phase (Fig. 2). Observation data suggest that EAV in wetland has a high light interception capacity by adjusting leaf phenotypes over seasons, which is an external manifestation of their strong carbon and nitrogen fixation capacity. This significant difference in LAD between the 2 species indicates the diversity of species phenotypes controlled by gene expression and environmental factors.

#### Vertical gradient of leaf normal inclination angle

Field observations revealed significant vertical and seasonal variation in the LAD of *P. australis* canopies. Interestingly, the leaf normal inclination angle varies only slightly with season but remains almost constant in the vertical direction of the canopy due to its unique leaf morphology. In the analysis, we focused solely on the vertical shift of *P. australis*. Figure 3 illustrates the seasonal shifts over the *P. australis* canopy from top to bottom.

Within the top layer of the canopy, species are predominantly erectophile or platiophile during spring and winter, respectively. A shift toward planophilia occurs between spring and summer, manifesting again in autumn and winter. Despite general consistency throughout the last 3 seasons, noticeable variations do exist, particularly with a higher incidence of vertically inclined leaves in summer and autumn, indicating more sunlight filtering into the understory. To sum up, leaf inclination decreases from spring to autumn and then increases in winter at the apex and base of the canopy. Leaf angle distribution (more vertical than horizontal) is instrumental in enhancing plant photosynthesis [25]. Each leaf has a distinct orientation that fluctuates across different layers [45]. Some plants even change leaf position within hours or minutes daily, helping to manage temperature and harness solar radiation.

Surprisingly, the LAD exhibits a complementary trend across the entire canopy height, optimizing sunlight interception across seasons. In short, from summer to winter, the horizontal distribution of leaf inclination angles at the bottom of the canopy was more extensive than in spring. Supporting this observation is the quantified mean tilt angle (MTA) (Table).

**Table.** Seasonal variations in mean tilt angle (MTA) of *P. australis*

Leaf layer	MTA $\left(\text{degrees}\right)$			
	DOY = 129	DOY = 256	DOY = 301	DOY = 355
Top	55.9	50.9	43.0	46.5
Middle	40.4	36.1	33.4	31.7
Bottom	32.0	22.9	17.2	18.4

The MTA of the canopy descended sequentially from top to bottom, showing an erectophile pattern, revealing that the leaf normal inclination angle shifted toward horizontality with increasing depth, culminating in a planophile distribution at the bottom. Analysis indicates that wetland EAV exhibits strong light absorption capabilities through seasonally and vertically regulated leaf morphologies.

*P. australis* enhances light interception by adjusting the leaf inclination angle of the canopy. This adjustment is simultaneously performed both seasonally and vertically to promote photosynthesis and accumulate biomass. The leaf layer at the bottom of the canopy enhances light capture by regulating leaf inclination angle distribution. *P. australis* extends the photosynthetic duration and prevents direct sunlight from burning at noon by varying the leaf inclination in vertical direction. Solar radiation hitting the bottom layer around noon heats the substrate beneath it; the stored thermal energy is then released when the solar elevation angle is reduced, further enhancing scattered radiation to the bottom leaf. Moist soil coupled with water matrix retains large amounts of heat for the canopy due to its higher heat capacity than dry soil.

#### Leaf area estimate

**Fig. 4.**
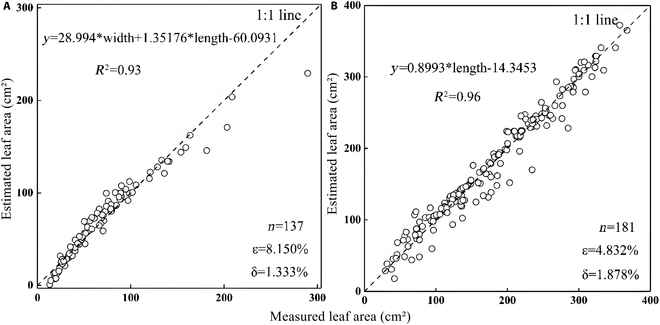
Comparison of measured and fitted values of leaf area. (A) Scatterplot of *P. australis* leaf area fitted from maximum leaf width and length versus measured values. (B) Scatterplot of estimated *T. orientalis* leaf area fitted from its length versus measured values.

Leaf shape is usually considered species specific, so leaf area can be expressed as the empirical relationship by length and width. After comparison, it was found that leaf area could be estimated using the binary linear regression model established by maximum leaf width and length (Fig. 4).

As shown above, the leaf area of *P. australis* can be simply represented by a binary linear equation of its maximum width and length. With a determination coefficient of 0.94, the linear estimation model has excellent prediction with only 1.333% model deviation, making it reliable for leaf area prediction. In the case of *T. orientalis* leaves, they are narrow and elongated, showing a strong correlation between their length and area. As shown in Fig. 4B, the determination coefficient between the estimated leaf length and the measured value can reach 0.96, indicating a precise model with a minimal deviation of only 1.878%.

#### Vertical heterogeneity of biochemical and structural parameters

Chlorophyll concentration in each leaf layer gradually increases with growth, peaking near the top layer excluding senescence. Similarly, xanthophyll follows a parallel path to chlorophyll, except during its peak in autumn. This indicates that xanthophyll cooperates with chlorophyll throughout plant growth. Leaf water indicators (leaf water concentration and equivalent water thickness) showed opposite trends during vigorous growth (peaked at the top layer) and senescence (accumulated at the bottom layer), indicating that water transferred from top to bottom as vegetation matured.

Specific leaf area (SLA), indicative of leaf surface area per unit dry weight [46], is directly influenced by leaf thickness, morphology, and weight. It symbolically mirrors the leaf’s potential for light capture and robust self-protection against intense light [47]. Therefore, SLA has indicative significance for determining the photosynthetic rate of vegetation. Seasonal dynamics of SLA and EWT are critical metrics for elucidating plant physiological status and adaptive capabilities. At the peak of summer growth, SLA exhibits a discernible upward gradient from top to bottom of the canopy, with EWT peaking simultaneously in the uppermost leaf. Conversely, as leaves transition through the senescence phase, SLA’s trajectory reverses, showing a rise from bottom to top canopy sections. Correspondingly, EWT masses are mainly in the bottom layer leaves during senescence. Notably, the consistency of these seasonal patterns between SLA and EWT is consistent with the transition pattern of biomass accumulation, initially concentrated in the bottom layer leaves and later shifting to the top canopy as leaves age. This coherence suggests a complex interaction between leaf physiology and plant growth strategies.

**Fig. 5.**
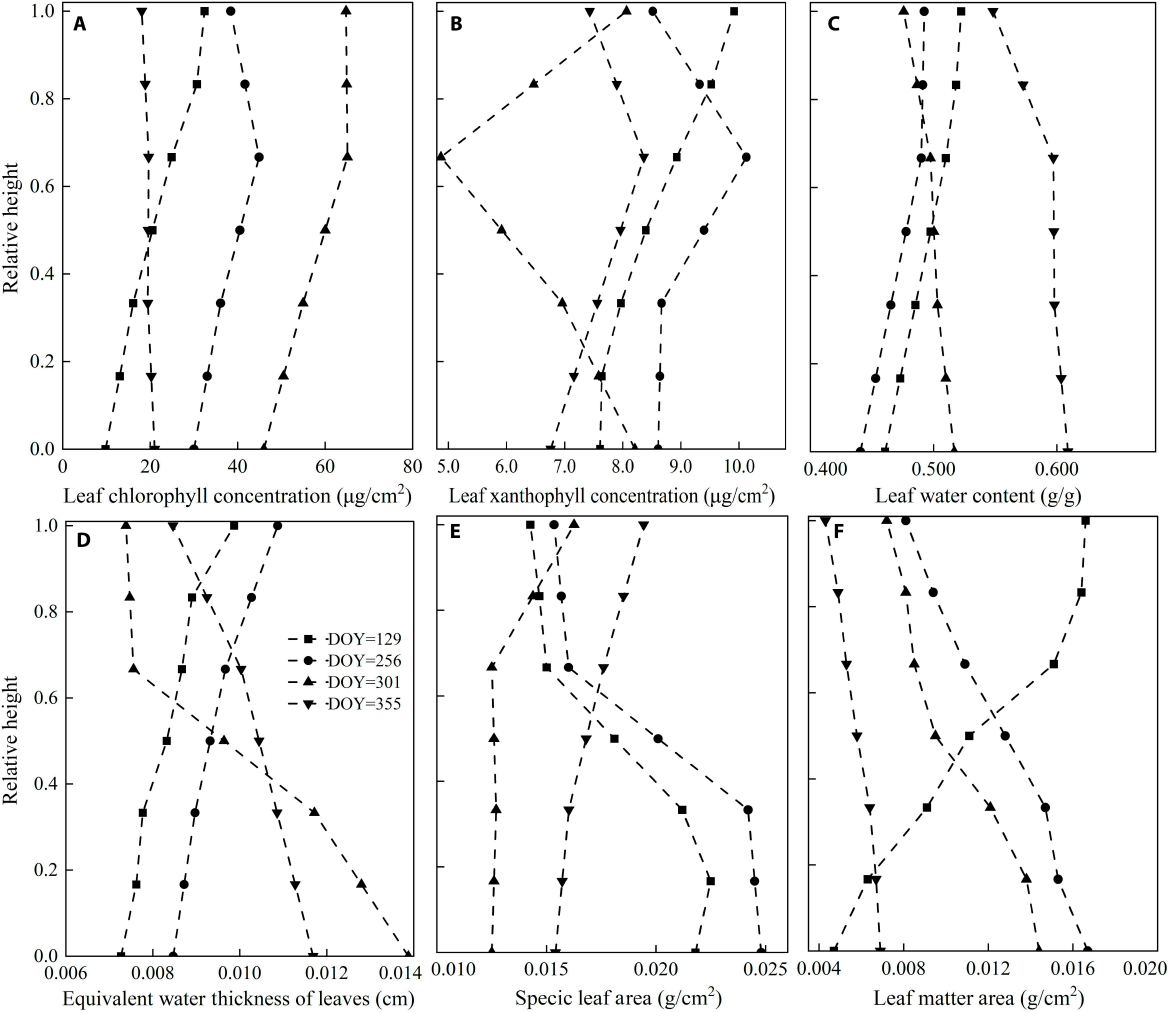
Vertical variation pattern of physiological and biochemical parameters of *P. australis*. (A) Leaf chlorophyll concentration. (B) Leaf xanthophyll concentration. (C) Leaf water concentration. (D) Equivalent water thickness. (E) SLA. (F) Leaf dry matter.

In the early stages of growth, an obvious trend emerges where the dry matter content of the leaf gradually diminishes as it descends through the canopy layers. However, this pattern reverses during the peak growth seasons of summer and autumn, when the content persists regardless of canopy depth, remaining even into the maturing period in winter. This reversal indicates that dry matter accumulation initially begins in the uppermost canopy leaves, gradually shifting to the lower layer (Fig. 5).

**Fig. 6.**
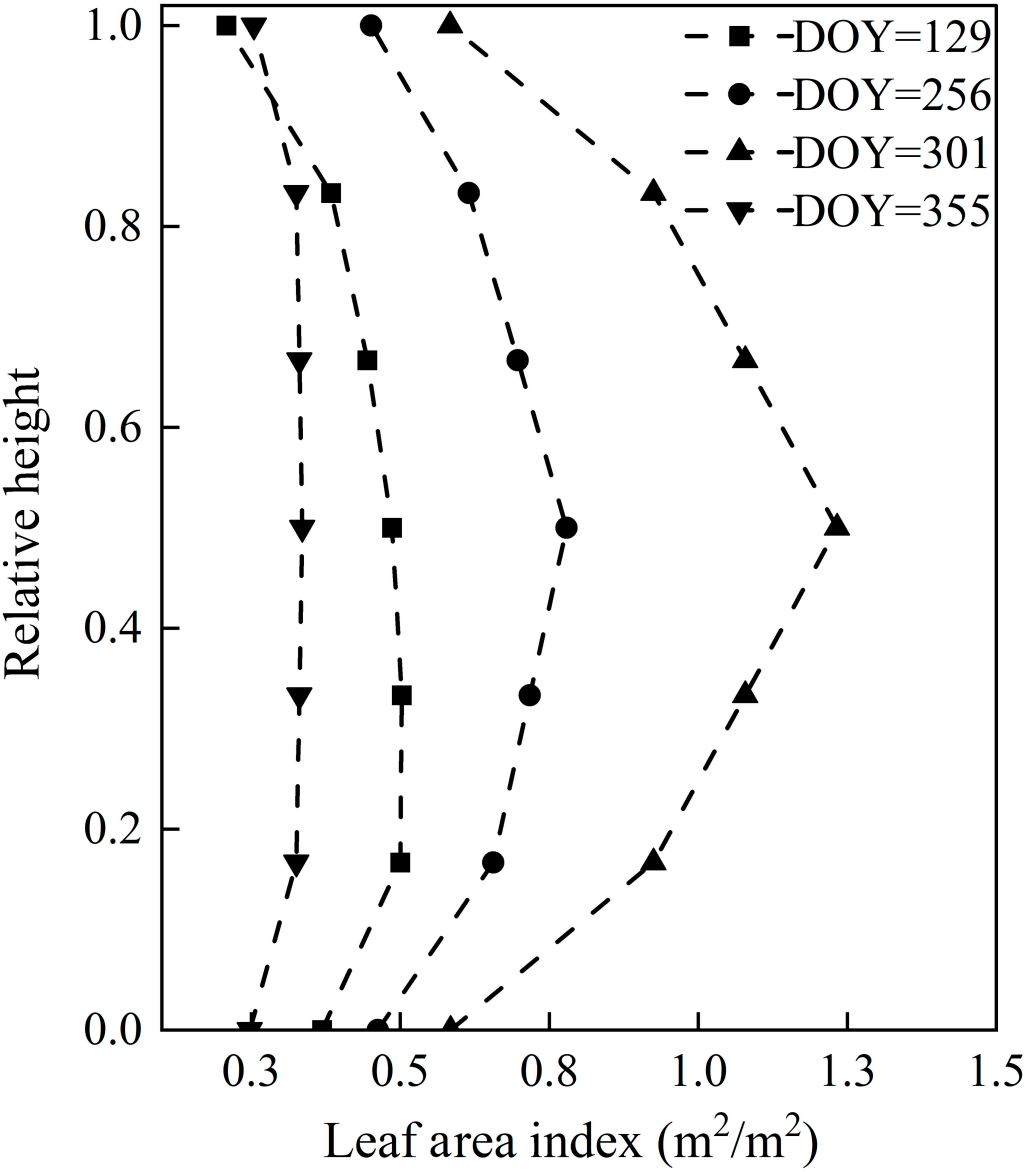
LAI vertical profile of *P. australis*.

Concerning the vertical configuration of LAI, a stratified distribution emerges, showing a marked peak in the middle layers and a distinct dip at the bottom. Specifically, the peak LAI value is reached at a leaf height of 0.50, with minimal differences observed between the highest and lowest leaf layers, which is mainly due to the increase in individual leaf areas. Remarkably, from the early stages of growth through the vigorous phases (DOY = 355), the leaves exhibit a remarkably uniform vertical pattern. Accumulation in the middle leaf layer occurs most rapidly, peaking in autumn and then decreasing rapidly with aging. During this phase, stratified LAI profiles maintain consistent homogeneity (Fig. 6). In addition, the LAI vertical profile pattern reveals that the canopy structure of *P. australis* communities can be represented as inverted triangular cones during modeling, validating Zhou et al.’s [48] model effectively.

### Canopy light radiation distribution pattern

The transmission characteristics of solar radiation in the cluster mainly depend on the structural characteristics of the cluster, such as the quantity of leaf and leaf layer elements, their size, spatial orientation, and dispersion within the cluster. Evaluating the mean projection and extinction coefficient of the unit leaf area in the vertical solar plane illuminates crucial insights into the attenuation of sunlight within the cluster. In addition, it is an indispensable parameter for calculating the canopy overlap function of each component in any spatial direction within the bidirectional reflectance model of *P. australis*. This allows accurate simulation of the “hot spot” effect displayed by the canopy at various orientations.

#### Leaf projection *G* function and canopy extinction coefficient

**Fig. 7.**
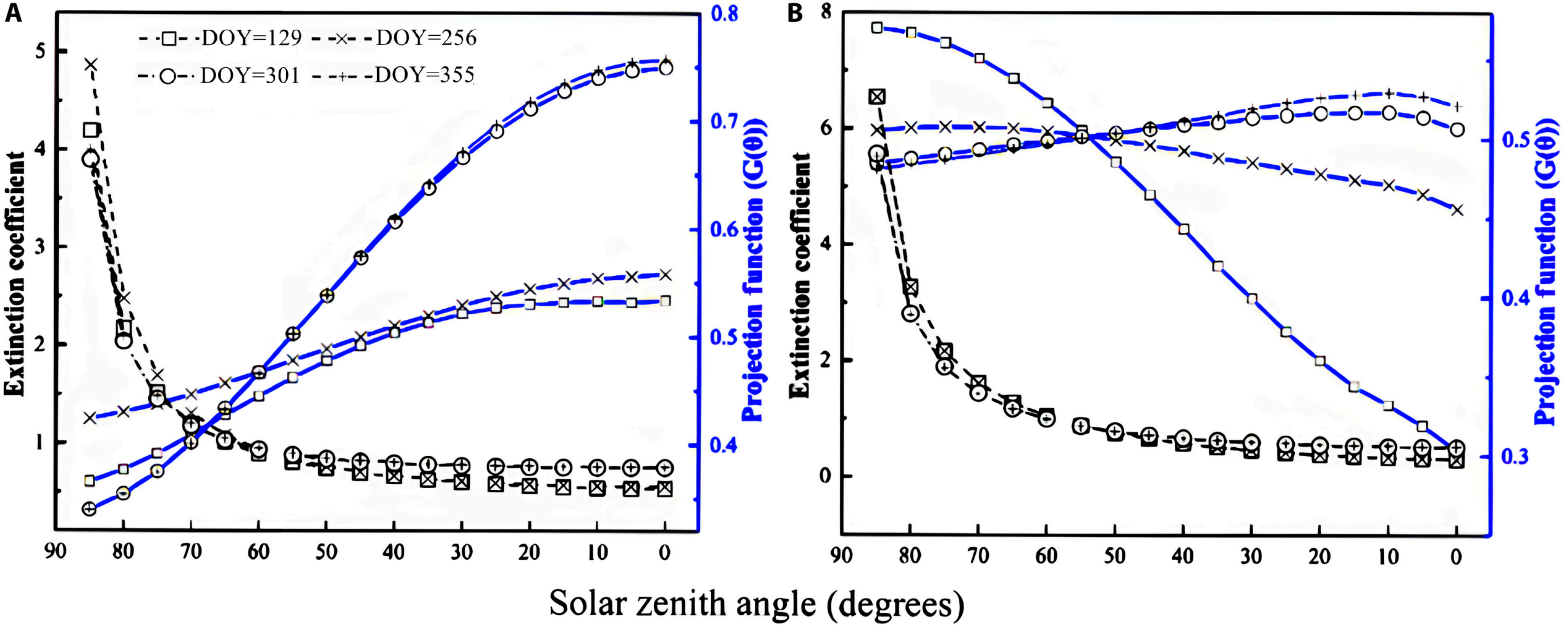
Canopy extinction coefficient and leaf projection *G* function against solar zenith angle (θ) of *P. australis* (A) and *T. orientalis* (B) in the principal phrase.

Seasonal variations in leaf projection function with respect to the solar zenith angle can be derived for both species according to the measured normal inclination angle of the leaf. As shown in Fig. 7, the canopy leaf projection functions of *P. australis* and *T. orientalis* exhibit significant seasonal changes due to variations in leaf inclination. Notably, the transformation of leaf projection function in the *P. australis* canopy across 4 seasons is mainly due to variations in leaf inclination angle, similar to the plagiophile pattern in spring and summer. In autumn and winter, the projection *G* function remains fairly uniform, notably distinct from that in spring and summer, perfectly aligned with the planophile pattern. On the other hand, the projection function of *T. orientalis* canopy leaves exhibits significant deviations from other seasons, in spring, resembling an extreophile pattern, which typically involves leaves adopting extreme positions. Despite minor differences, the projection functions in the other 3 seasons throughout the year show similarities to the plagiophile pattern.

**Fig. 8.**
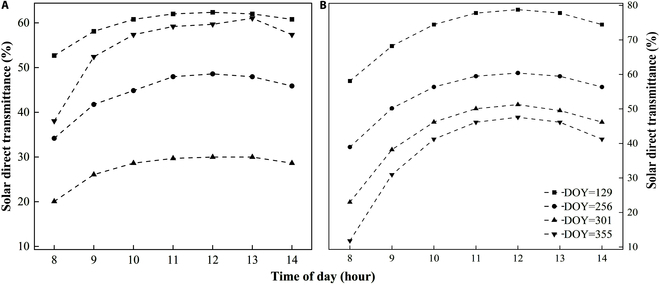
Distribution of solar direct radiation transmittance with season for *P. australis* (A) and *T. orientalis* (B). The horizontal axis is displayed as the observation time of day.

Despite the different leaf inclinations exhibited by the 2 species and their significant seasonal variations, their canopy extinction coefficients exhibited similar patterns of variation with the solar zenith angle across different seasons, differing only subtly in amplitude. Notably, when the solar zenith angle exceeded 70°, the extinction coefficient *k* underwent a steep increase, suggesting that the primary factor influencing the canopy extinction coefficient is variation in the solar zenith angle. During summer in the experimental area, the solar zenith angle typically ranged from 20° to 70°, with a *G* function value ranging from 0.53 to 0.40 and an extinction coefficient *k* ranging from 0.57 to 1.20.

Compared to the *P. australis* canopy, the extinction coefficient of the *T. orientalis* canopy, which features more vertical leaf angles, is notably higher than that of *P. australis* when the solar zenith angle exceeds 65°. This is consistent with the observation that as the solar elevation angle drops below 25°, canopies with an extremophile pattern intercept more horizontal light. This means that vegetation with more vertically distributed leaf angles can begin photosynthesis even when the solar elevation angle is small, such as during the early morning and just before sunset. Consequently, this, coupled with Earth’s rotation, extends the duration of effective photosynthesis throughout the day, allowing for greater accumulation of dry matter. In addition, a smaller canopy extinction coefficient helps to avoid direct midday sunlight (when the solar zenith angle is typically less than 30°), acting as a self-protection mechanism for plants. This adaptation could potentially explain the strong nitrogen fixation capacity observed in wetland EAV, revealing one of its mysteries.

#### Seasonal and daily variation of solar direct radiation transmittance

Applying Beer–Lambert law to data collected from 2020 to 2022 reveals that transmittance variations across *P. australis* clusters with respect to height follow a uniform pattern. Specifically, the top and bottom sections of the cluster exhibit a lower direct solar transmittance compared to the middle layer. This difference is attributed in particular to the increased density of *P. australis* leaves in the mid-section of the cluster, which effectively intercepts and scatters more solar radiation. This pattern suggests that the cluster’s midsection acts as a primary barrier to solar radiation, reducing the transmittance of light to the upper and lower layers (Fig. 8).

The direct solar radiation transmittance of *P. australis* clusters typically exhibits an inverse bowl shape, mainly influenced by cluster structure, with the greatest difference occurring during the initial sunrise phase (8 to 10 points). In terms of amplitude, the transmittance peak occurs in the early growth phase and diminishes in the vigorous growth phase, mainly due to the increase of LAI with *P. australis* growth. The *P. australis* cluster exhibits a consistent overall shape in the solar direct radiation transmittance change curve except during the aging phase, indicative of vertical leaf inclination and density adjustments to ensure uniform light exposure across the canopy leaves.

The normal leaf inclination of *T. orientalis* exceeds that of *P. australis*, showing an inverse “V” curve pattern in solar direct radiation transmittance. Spring records the highest values, followed by summer, and winter shows the lowest. This change is mainly driven by LAI and seasonal variations in solar zenith angle. Unlike *P. australis*, *T. orientalis* maintains similar trends in direct solar radiation transmittance regardless of season, due to the constant normalized leaf inclination. The LAI escalates with an increase in leaf density, increasing the leaf layer’s ability to capture light, and ultimately lowering canopy transmittance.

Variations in leaf inclination angle and leaf density vertically within the canopy dictate the absorption of light energy and the direct transmission of solar radiation across its layers. Although the leaves of the *P. australis* canopy are most densely concentrated in the middle, the volume at the top and bottom of the layer is comparable to that in the middle. When a layer of equal thickness is penetrated by light, each leaf layer transmits light similarly. The canopy is represented as an ellipsoid, and the volume density of the internal leaf area is uniform across it. However, due to small canopy volumes at the top and bottom, the low LAI results in greater direct solar radiation transmittance.

#### Vertical distribution pattern of solar direct radiation transmittance

**Fig. 9.**
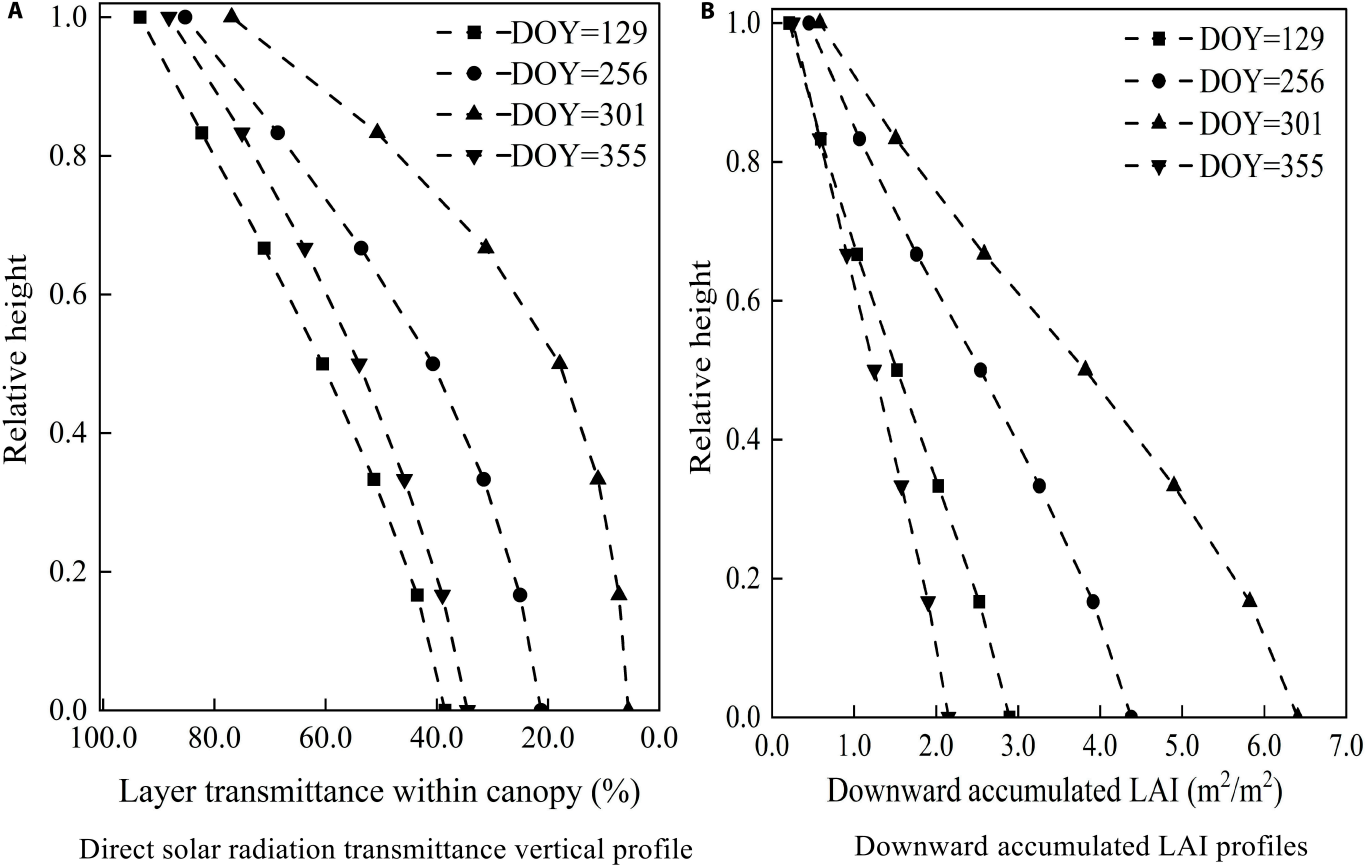
Vertical distribution pattern of direct solar radiation transmittance and downward cumulative LAI in the *P. australis* canopy. (A) Direct solar radiation transmittance vertical profile. (B) Downward accumulated LAI profiles. The smooth curve is fitted to the transmittance of each layer of leaves to obtain the vertical profile of their transmittance. The solar zenith angle at observation time is the angle corresponding to 10 o’clock.

Vertical transmittance distribution patterns of *P. australis* at different growth stages (Fig. 9) were fitted using linear curves. Due to its consistent inclination angle, solar radiation transmission in *T. orientalis* canopy is primarily influenced by solar elevation angles; therefore, additional analysis is unnecessary.

As shown in Fig. 9, except for the top and bottom of the canopy, the transmittance inside the canopy did not show a significant gradient in the vertical direction. Over the 4 seasons, the transmittance in the *P. australis* cluster gradient decreases as the relative canopy height increases. The direct solar radiation transmittance at the top layer is basically equal to that at the bottom layer of the cluster and is largest at the middle layer. This distribution characteristic is obviously due to the concentrated distribution of *P. australis* leaves at the middle layer of the cluster, which conforms to the light intensity distribution law of the vegetation cluster.

Nevertheless, the distribution of *T. orientalis* leaves in clusters ensures that the inclination angle of the leaves remains constant regardless of the height of the canopy. As a result, direct solar radiation transmittance is uniform throughout, except for the root area within the canopy. No pattern of vertical variation in transmittance was observed. The uniform distribution of direct solar radiation transmission within the leaf layer enhances light energy utilization, minimizes leaf-to-leaf competition, mitigates temperature gradients, and stabilizes plant structure. The equal dispersion of direct solar radiation transmission between leaf layers ensures adequate light energy absorption by leaves and reducing interlayer light energy competition, thereby boosting light energy utilization efficiency and organic matter accumulation and enhancing plant growth and expansion.

### Vertical associations among stratified parameters during growth

**Fig. 10.**
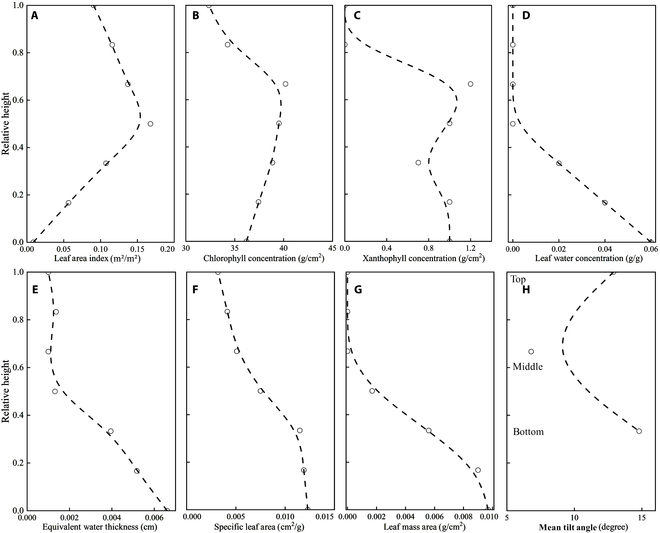
Vertical difference in biochemical and structural parameters of the *P. australis* canopy during growth stage. (A) LAI. (B) Leaf chlorophyll concentration. (C) Leaf xanthophyll concentration. (D) Leaf water content. (E) Equivalent water thickness. (F) SLA. (G) Leaf matter area. (H) Leaf normal inclination angle. The smooth curve is fitted by B-spline curve to show the trend of parameter changes along canopy height.

The above results show the vertical heterogeneity of leaf biochemical and structural parameters during the growth stage. Associations between them and how they jointly affect plant growth and ecological functions are interesting. The difference between the maximum value during the growth period (excluding winter) and spring will be used as an indicator to highlight the trend of changes in various parameters along the direction of canopy height during the growth period, as shown in Fig. 10.

From Fig. 10, it can be observed that during the growth process, the biochemical and structural parameters of *P. australis* increase significantly. However, the magnitude of changes across different heights showed varied patterns: LAI and leaf chlorophyll content increased most in the middle of the canopy, while the increase in leaf xanthophyll content was mainly concentrated in the middle and bottom layers, almost unchanged at the top layer. Leaf water concentration, equivalent water thickness, SLA, and dry matter content increased most at the bottom of the plant, indicating that biomass accumulation was highest in the middle and bottom layers. The mean tile angle of leaf changes the most at the bottom of the plant, followed by the top, with the smallest increase in the middle. Overall, the biochemical and structural parameters of each leaf layer showed a similar trend of change.

The normalized leaf inclination angle shows an opposite trend to the stratified LAI, which may indicate that a stable leaf inclination angle is beneficial for increasing the LAI. The parameters that characterize leaf water concentration (equivalent water thickness and leaf water concentration) are consistent with the structural parameters that characterize biomass (SLA, leaf dry matter content), indicating that organic matter increases biomass as water is transferred to the bottom of the canopy. Considering that the bottom leaves of the *P. australis* canopy are closest to the underlying surface of the canopy, the water content of the bottom leaves increases the most, and vegetation transpiration is generally strongest in the top leaves. This may suggest a strong transpiration between the water vapor interface between the bottom leaves and the bottom of the canopy. More targeted observation data are needed to further verify this hypothesis.

## Discussion

The structure of vegetation association is the external manifestation of its adaptation to the environment. Preliminary analysis of in situ observation data reveals that wetland EAV is mainly affected by leaf element size, leaf number, leaf inclination angle distribution, and vertical heterogeneity. The heterogeneity of the vertical structure of the canopy determines the difference in the distribution pattern of direct solar radiation energy within the association. This difference in the size and quantity of leaf elements is finally reflected by the LAI, as the distribution of leaf inclination and LAI directly affects the redistribution of direct solar radiation energy within the group. Therefore, LAD and direct solar radiation of typical wetland vegetation are further discussed.

### Daily changes in direct solar transmittance within the canopy

**Fig. 11.**
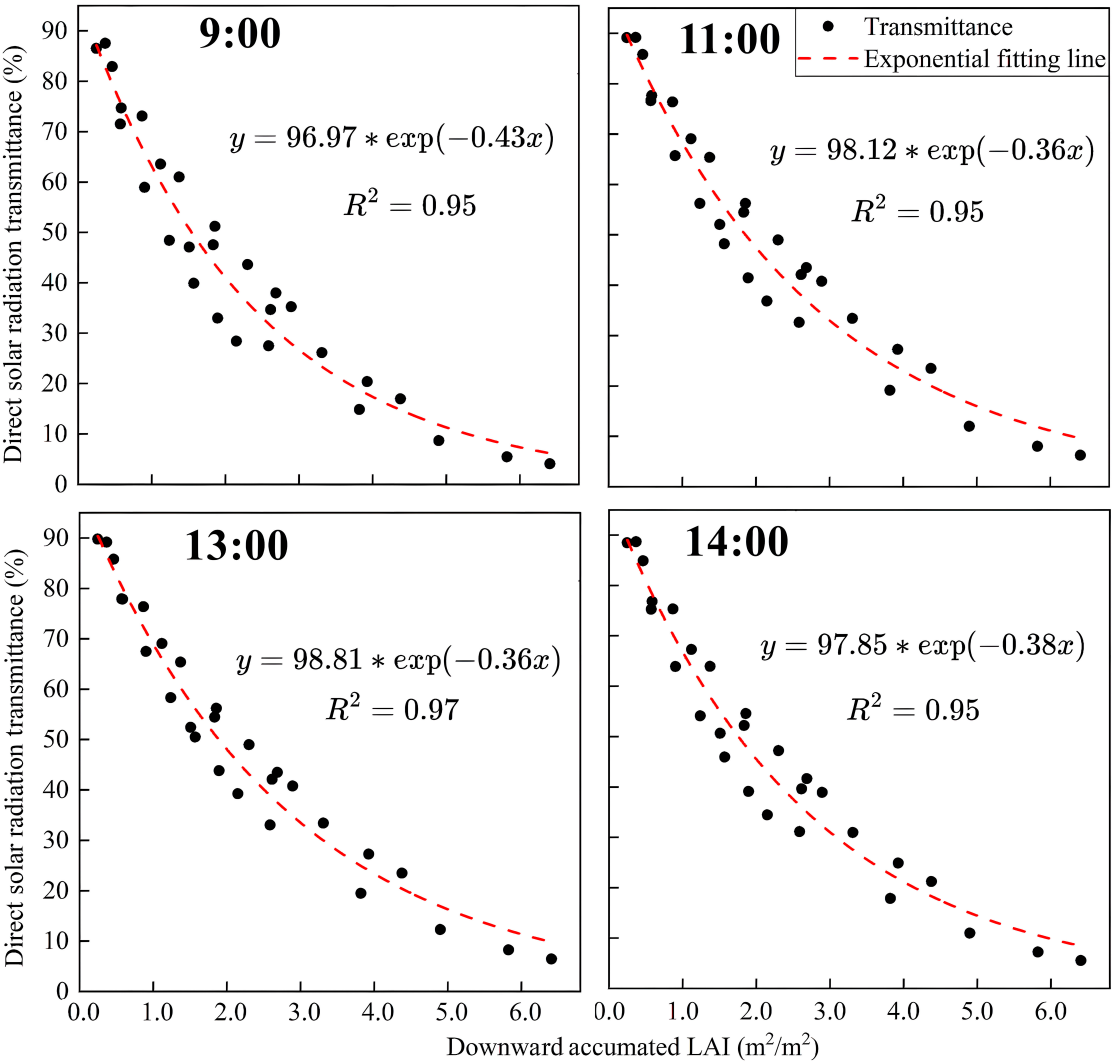
Exponential fitting curve of direct solar radiation transmittance and downward cumulative LAI in the *P. australis* canopy at different time. The smooth curve is fitted to the transmittance of each time of the day to obtain their transmittance.

Direct solar radiation transmittance shows a very similar decreasing trend at different canopy heights and time periods, accompanied by an increase in the cumulative decrease in LAI. Average direct solar radiation transmittance in the upper part of the canopy (above heights with accumulated LAI less than 1.4) is relatively high. From the top of the canopy downward, it decreases rapidly with the increase of accumulated LAI. Average direct solar radiation transmittance near the bottom of the canopy (below heights with accumulated LAI greater than 4.6) is very low, and there is little variation between treatments. Within the canopy, there is a significant exponential decrease in direct solar radiation transmittance as accumulated LAI increases. This trend can be quantitatively described by the exponential function. The deterministic coefficients of the fitted equation consistently exceed 0.95 (Fig. 11). The canopy extinction coefficient (*K*), shown to vary diurnally, is smallest at midday and largest at dawn/dusk. Species with erect leaf inclination angles exhibit reduced *K* values, thus enhancing their light-harvesting potential. Similar findings have also been confirmed related to terrestrial vegetation and crops [49,50].

### Stratified radiation patterns versus biochemical–structural parameters

Previously, our findings elucidated the link between direct solar radiation transmission and cumulative LAI within the canopy. These observations are consistent with Beer’s law, but canopy pigment concentration and structural variables are more significant and of interest, such as canopy chlorophyll concentration, canopy equivalent water thickness, and canopy dry matter content. At a specific depth, canopy pigment concentration equals the product of leaf pigment concentration and LAI of the same sublayer. This section examines the correlation between direct solar radiation transmission and canopy parameters across different canopy depths and seasonal periods.

**Fig. 12.**
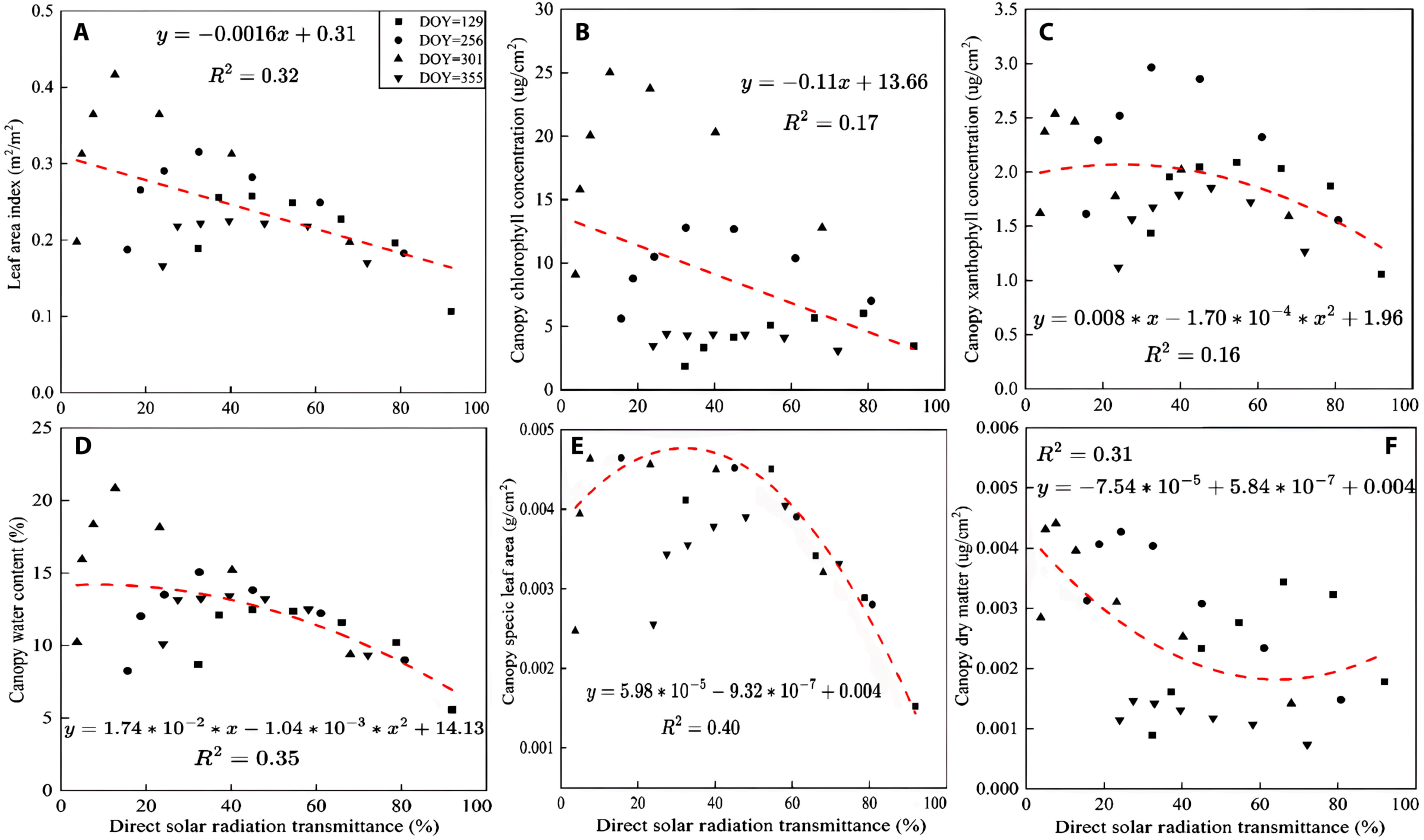
Correlation analysis between direct solar radiation transmittance and stratified canopy structure and pigment parameters of *P. australis*. (A) LAI. (B) Canopy chlorophyll concentration. (C) Canopy xanthophyll concentration. (D) Canopy water content. (E) Canopy SLA. (F) Canopy dry matter.

In the previous section, we discussed that direct solar radiation transmittance and cumulative LAI conform to the law of light attenuation. Notably, the layered LAI did not strictly follow the law at specific depths (Fig. 12A). However, seasonally, there is a strong negative correlation, suggesting the potential for estimating the layered LAI through direct solar radiation transmittance. Significant negative correlations were also noted between direct solar radiation transmittance and canopy structural parameters: LAI, dry matter content, SLA, and water concentration (absolute correlation coefficients are all greater than 0.55). Interestingly, the correlation with canopy chlorophyll and xanthophyll concentrations was relatively weak (negative correlation coefficients are all less than 0.4), in contrast to structural parameters. Luo et al. [50] provided direct evidence of the correlation between these parameters in sugarcane, but no further focused discussion.

The above analysis shows poor correlation between canopy pigment parameters and direct solar radiation transmittance excluding LAI impact. Therefore, the sensitivity of direct solar radiation transmittance to canopy structural parameters exceeds significantly that of canopy pigment parameters, suggesting the potential to estimate vegetation canopy layering structure parameters based on direct solar radiation transmittance of layered solar radiation (Fig. 12).

### Estimation of stratified direct solar transmittance

The analysis indicates that direct layered solar radiation transmission within the canopy correlates with canopy structural parameters. Therefore, accurate quantification of this transmission using the LAI of canopies is crucial for determining the layered structure of the canopy.

The canopy cluster is equally divided into *N* layers (Eq. 2) and each layer is statistically independent, where *L* is the total LAI and ∆L is the LAI of each layer. Thus, the direct sunlight transmittance within the cluster can be expressed as Eq. 3, where *K* is the canopy extinction coefficient. By substituting *n* into the above equation, Eq. 8 can be obtained and transmittance for each layer from the top to bottom leaf canopy is calculated by Eq. 8 as follows:

$$T={\left(1+\frac{K}{N}\right)}^{-N}$$ (8)

**Fig. 13.**
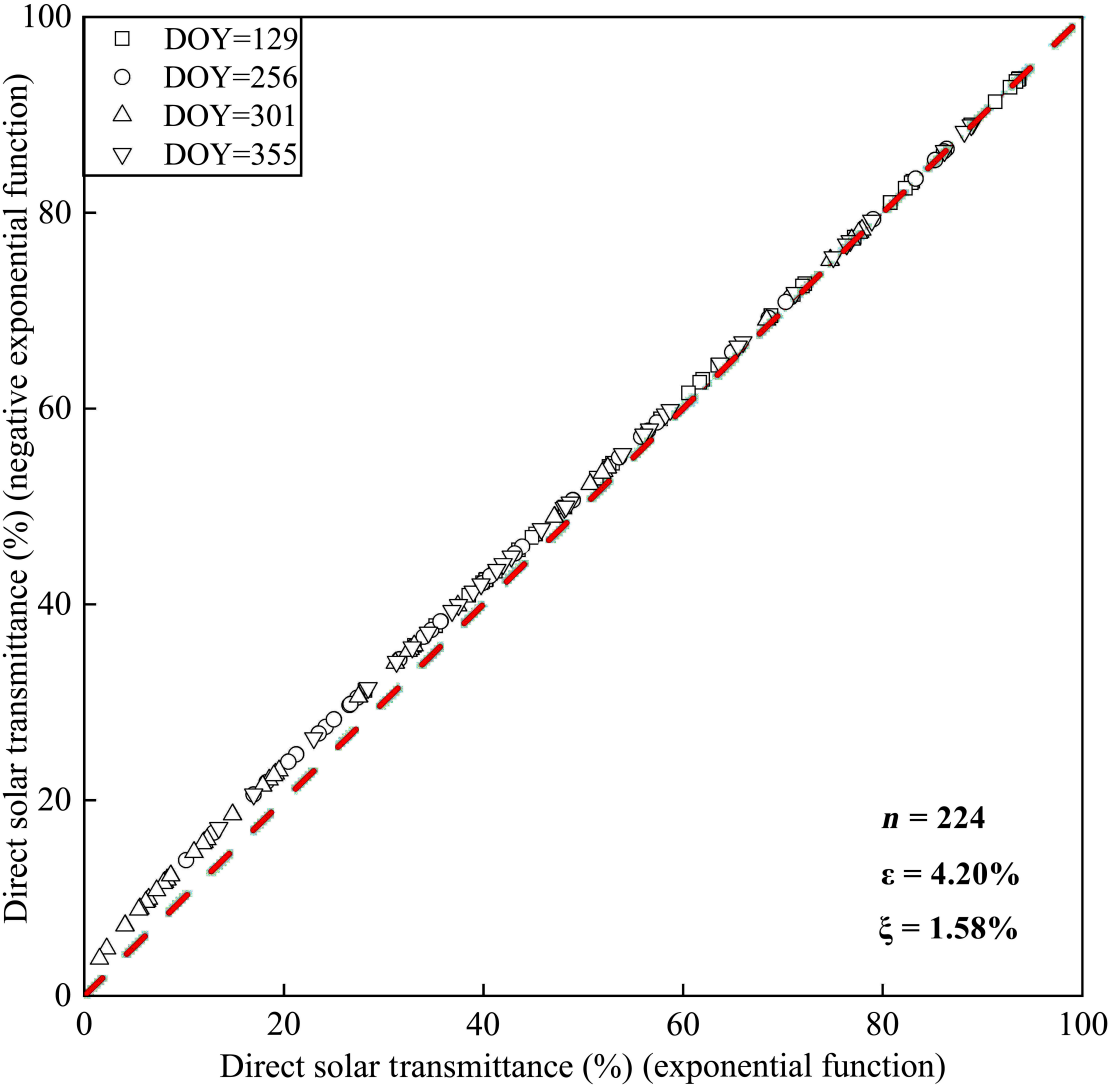
Comparison of layered solar direct radiation transmittance driven by exponential function and negative binomial function in different growth stages.

As shown in Fig. 13, the layered solar direct radiation transmittance driven by negative binomial functions at different growth stages is very close to the values calculated by exponential functions, and the overall deviation between the 2 is less than 2%, as shown in Fig. 13, which is consistent with Shen et al. [49]. Due to the convergence of Eq. 8, its limit converges to $T=e^{-K*L}$, which happens to be the expression of Beer’s law. Therefore, the transmittance within the canopy can be approximately calculated by Eq. 8 (Fig. 13).

We calculated and evaluated the direct layered solar radiation transmittance of *P. australis* at 8:00 AM to 3:00 PM in different seasons by hourly results. The coefficient of determination, with a value of 0.99, indicates that the estimation model has good prediction accuracy with only 4.20% model deviation, and a positive deviation of 1.58%, making it reliable for layered LAI prediction. The formulation’s deduction implies that in the absence of specific information on the layered LAI or the cumulated LAI, an approximate calculation for stratified transmittance within the canopy could use a negative binomial function incorporating both the total LAI and the number of layers [40,49].

### Limitations and future research

This paper, focusing on aquatic plants in wetlands, demonstrated that direct layered solar radiation transmittance in estimating canopy stratified structure parameters. Further validation is needed for other wetland aquatic vegetation such as mangroves. Given the limited penetrability of light in dense vegetative clusters, coupling active and passive remote sensing techniques appears promising, such as using LiDAR (light detection and ranging) to extract vertical profiles of canopy structure, and associating optical satellites for precise monitoring of the fine structure of wetland aquatic vegetation. Therefore, a radiation transfer model incorporating active and passive remote sensing, and taking into account the vertical heterogeneity of vegetation parameters, is a promising method for monitoring vegetation ecological processes at finer scales. In addition, to be honest, it should be noted that vegetation fine structure stratification schemes vary across fields due to different perspectives. This paper focuses on remote sensing modeling, which requires further research into more reasonable layering and layer numbers.

## Conclusion

Delineating a dynamic light radiation distribution pattern within the EAV canopy based on Beer–Lambert law coupled with radiative transfer theory was examined in this study. By adjusting the vertical distribution patterns of leaf normal inclination angle, LAI, *P. australis* makes the direct solar radiation transmittance at each leaf layer gradient decrease, while the vertical distribution pattern of the direct solar radiation transmittance *T. orientalis* canopy is only affected by leaf normal inclination and incident solar radiation. Additionally, *P. australis* modulates the vertical distribution of physiological, biochemical, and structural attributes, including normalized leaf inclination, throughout various growth stages, ensuring a uniform distribution of solar radiation in the canopy for enhanced canopy light interception. *P. australis* and *T. orientalis* display vertical phenotypic diversity within a consistent growth environment, exhibiting contrasting photonic light energy regulatory strategies driven by biodiversity.

Compared to pigments, the layered transmittance of direct solar radiation appears to be more correlated by layered canopy structural elements. The negative binomial function simulated layered direct solar transmittance with a deviation of less than 2%, suggesting considerable potential for our methodology in estimating stratified biochemical–structural parameters. Integrated active and passive remote sensing radiative transfer models accounting for vertical vegetation complexity are anticipated to enhance fine-scale monitoring of vegetation ecological functions, serving as a key initiative for our future endeavors.

## Appendix A. Leaf normal inclination angle distribution, projection function, and extinction

The leaf normal inclination angle denotes the angle between the leaf surface and the normal direction of leaf surface. Numerous studies have validated the superior capability of the trigonometry algorithm in determining the leaf angle of the erectophile pattern [34,36]. Specifically, the trigonometry algorithm employs a linear orchestration of trigonometric functions to fit the leaf angle distribution, expressed as:

$$x=2∙\theta_{L}$$ (9)

$$y=a\text{sin}\;x+\frac{b\text{sin}\;2x}{2}$$ (10)

$$∆x=\left(y-x+2∙\theta_{L}\right)/2$$ (11)

x=x+∆x (12)

$$\text{Until}:\left|∆x\right|<t$$ (13)

$$F\left(\theta_{L}\right)=2*\left(y+\theta_{L}\right)/\pi$$ (14)

For a given $\theta_{L}$determined in radians, $F\left(\theta_{L}\right)$ can be calculated via rapid iteration employing Eqs. 11, 13, and 14, Here, *t* denotes a suitable threshold, e.g., 10^−6^, *a* and *b* are 2 parameters that need to be optimized, and x and y pertain to the cumulative leaf inclination distribution $F\left(\theta_{L}\right)$, which is the fraction of leaf area with an inclination below. For further details on this algorithm, refer to Verhoef [36]. We executed meticulous experiments and deduced optimal parameters for the 2 species described in this paper [34].

The leaf projection function $G\left(\theta \right)$ describes the absorption, reflection, or transmission of solar radiation by individual leaves within a canopy. Given an azimuthally symmetric canopy, the leaf projection function $G\left(\theta \right)$ quantifies the unit leaf area’s projection coefficient on a plane perpendicular to the viewing direction [30]. Typically, the *G*(*θ*) function is written in terms of the angle *θ* between the incident light and observation directions. It is calculated as:

$$G\left(\theta \right)=\int_{0}^{\pi /2}A\left(\theta ,\theta_{L}\right)f\left(\theta_{L}\right)d\theta_{L}$$ (15)

$$\begin{array}{c} A\left(\theta ,\theta_{L}\right)=\left\{\begin{array}{cc} \text{cos}\theta \text{cos}\theta_{L} & \left|\text{cot}\theta \text{cot}\theta_{L}\right|>1\\ \text{cos}\theta \text{cos}\theta_{L}\left[1+\left(2/\pi \right)\left(\text{tan}\vartheta -\vartheta \right)\right] & \left|\text{cot}\theta \text{cot}\theta_{L}\right|\leq 1 \end{array}\right. \end{array}$$ (16)

$$\varphi =\text{cos}\theta^{-1}\left(\text{cot}\theta \text{cot}\theta_{L}\right)$$ (17)

where *A* is the projection coefficient for the leaf inclination angle $\theta_{L}$ and the view zenith angle θ. Values for $G\left(\theta \right)$ are confined within 0 and 1.0; generally, they approximate 0.5 for all $f\left(\theta_{L}\right)$ when θ is 1 radian (≈57.3°) [30,36,37].

The canopy extinction coefficient (K) elucidates how much light permeates through the canopy and how it diminishes to the ground secondary to absorption, reflection, and scattering by leaf and other canopy components. The extinction coefficient is employed to depict the attenuation trend of solar radiation by plant communities. $K\left(\theta ,\theta_{L}\right)$ is affected by many factors including plant density, leaf number, leaf orientation, leaf arrangement, leaf area size, leaf layer thickness, chloroplast content, chloroplast arrangement, leaf surface smoothness, and light wavelength. According to the principle of solar radiation transmission, it can be expressed as follows:

$$K\left(\theta ,\theta_{L}\right)=G\left(\theta ,\theta_{L}\right)/\text{cos}\theta$$ (18)

where θ is the solar zenith angle and $\theta_{L}$ is the leaf normal inclination angle in radian. A high extinction coefficient indicates that the canopy intercepts more solar radiation.

## Data Availability

Data will be made available on request.
